# Fabrication of 3D Printed Ceramic Part Using Photo-Polymerization Process

**DOI:** 10.3390/polym15071601

**Published:** 2023-03-23

**Authors:** Da-Sol Lim, Jin-Kyo Chung, Ji-Sun Yun, Min-Soo Park

**Affiliations:** 1 Department of Mechanical System Design Engineering, Seoul National University of Science and Technology, 232 Gongneung-ro, Nowon-gu, Seoul 01811, Republic of Korea; 2 Department of Mechanical Information Engineering, Seoul National University of Science and Technology, 232 Gongneung-ro, Nowon-gu, Seoul 01811, Republic of Korea; 3 Energy & Environmental Division, Korea Institute of Ceramic Engineering and Technology, 101, Soho-ro, Jinju 52851, Republic of Korea

**Keywords:** photo-polymerization, 3D printing, ceramic, high viscosity, sintering, patterned curing

## Abstract

Ceramics are high-strength and high-temperature resistant materials that are used in various functional parts. However, due to the high strength and brittleness properties, there are many difficulties in the fabrication of complex shapes. Therefore, there are many studies related to the fabrication of ceramic parts using 3D printing technology optimized for complex shapes. Among them, studies using photo-polymerization (PP) 3D printing technology with excellent dimensional accuracy and surface quality have received the most widespread attention. To secure the physical properties of sintered ceramic, the content and distribution of materials are important. This study suggests a novel 3D printing process based on a high-viscosity composite resin that maximizes the content of zirconia ceramics. For reliable printing, the developed 3D printers that can adjust the process environment were used. To minimize warpage and delamination, the divided micro square pattern images were irradiated in two separate intervals of 1.6 s each while maintaining the internal chamber temperature at 40 °C. This contributed to improved stability and density of the sintered structures. Ultimately, the ceramic parts with a Vickers hardness of 12.2 GPa and a relative density of over 95% were able to be fabricated based on a high-viscosity resin with 25,000 cps.

## 1. Introduction

Ceramics are high-strength and high-temperature-resistant materials that are widely used in automobiles, electronics, energy components, various machinery parts, jewelry, and bio industries [1,2,3,4]. Especially in the medical field, ceramics are replacing parts of the body, such as artificial bones and teeth, due to their high biocompatibility [5]. However, ceramics have a higher strength than metals, making them difficult to machine in general. In addition, it is difficult to produce complex and various shapes because it is a brittle material. In order to overcome these limitations of ceramic processing, there is a growing body of research on the fabrication of ceramic structures using 3D printing, an additive manufacturing (AM) method. When 3D printing technology is used for fabricating ceramic structures, it is possible to create complex three-dimensional shapes easily.

The use of 3D printing technology is widespread in the production of various prototypes and special-purpose parts. Its advantage lies in the ability to manufacture parts with complex structures into three-dimensional shapes simply by designing them [6,7]. Consequently, the application areas of 3D printing technology are gradually expanding, and its importance is increasing in various industries, such as mechanics, architecture, electronics, and medical care [8,9,10,11]. As the scope of applications of 3D printing technology is gradually increasing, attempts are being actively made to manufacture structures that can actually be used based on various materials beyond simple shape production. One of the most promising areas is 3D printing technology based on ceramic materials with a wide range of functionalities. Ceramic 3D printing technology is an area of research with tremendous potential, particularly due to its ability to produce functional parts with complex structures that are challenging to manufacture using traditional methods [12].

Currently, the main methods for printing ceramic parts using 3D printing are binder jetting (BJ), material extrusion (ME), and photo-polymerization (PP). Since the BJ method is originally a powder-based process, it is easy to apply ceramic materials to commercial equipment, so it has been widely used since the beginning of related research. In 2016, a research team at The University of Texas at El Paso produced a ceramic structure using about 60% Al_2_O_3_ and 40% binder [13]. After sintering, the printed structure had physical properties of 96% relative density and 1.51 GPa hardness. However, in the case of the BJ method, there is a limitation in that the printed surface is rough, and the precision is low because the adhesive must be used. In addition, the hardness of the sintered structure was measured to be very low, on the order of 10% of commercial Al_2_O_3_, due to the low internal density during printing. Next, the ME method has the advantage that the structure of the equipment is simple and inexpensive. Extrusion using filaments or pastes mixed with ceramics and polymers is commonly used to produce ceramic parts in the ME method. In 2018, a study conducted at the Karlsruhe Institute of Technology utilized filaments composed of a mixture of Al_2_O_3_, LDPE, and paraffin to create structures with ceramic contents of up to 60 vol%. This structure had a relative density of 98.4% after sintering [14]. However, when printing in this manner, not only does the low precision suffer due to the flow characteristics of the extruded material during printing, but also the size limitations of the nozzle may make it impossible to create intricate shapes. Additionally, in the case of the ME method, the nozzle may wear due to the ceramic powder, which can cause issues when using the nozzle over a prolonged period.

The BJ and ME methods were widely used in the early stages of ceramic 3D printing research because of their relatively simple equipment configuration and printing methods. However, due to the limitation of precision, many attempts using the PP method have been made in recent studies. In 2018, a research group at Guangdong University of Technology made a photocurable resin containing approximately 20 vol% ZrO_2_ powder to produce a printed specimen with a Vickers hardness of 12–13 GPa [15]. The PP method can overcome the limitations of the BJ and ME methods in terms of precision and size, so recent ceramic 3D printing research has mainly utilized the PP method [16,17,18].

For these PP printers, resin supply/flattenation and photocuring reactivity are critical as the printing is based on the solidification reaction of the liquid resin. For this reason, most commercial resins are formulated with low viscosity and high flowability for ease of processing. However, when the ceramic powder is added, the viscosity of the mixture increases significantly in proportion to the amount of powder added. Therefore, in order to enable printing from commercial equipment, most of the early studies were limited to low levels of ceramic content. In such cases, it becomes difficult for the ceramic powders to bond during the sintering process, leading to reduced strength and density of the structure and resulting in numerous cracks and shape distortion. To overcome this content issue, recent studies have added various additives to increase the content to 56 vol% while maintaining a viscosity below 10,000 cps [19]. The high ceramic content green body was successfully printed and sintered, resulting in the fabrication of a structure of excellent quality. However, it can be seen that a lot of additives have to be added to reduce the viscosity, and there are still constraints on the powder content. Recently, some researchers have developed a ceramic 3D printing process based on high-viscosity composite resins to overcome some of the limitations of PP-type ceramic 3D printing. When the high-viscosity ceramic was used, it was possible to increase the content and overcome the sedimentation problem caused by the difference in specific gravity between materials [20,21]. However, due to the high viscosity, the fluidity of the resin was rapidly lowered, making it difficult to flatten and apply uniformly. In addition, photo-curability was lowered due to the increased viscosity, so the irradiated UV power should be increased. This could lead to warpage and cracks due to thermal shock and shrinkage during the curing reaction. In order to overcome this problem, in this study, a PP-type 3D printer capable of controlling various environments, such as temperature, was produced by the lab itself, and various process change experiments were conducted. After sintering, the fabricated structures will be tested for properties such as relative density and hardness to determine their potential for use as ceramic components.

## 2. Materials and Methods

### 2.1. Photo-Polymerization of Ceramic Composite Resin

**Figure 1:**
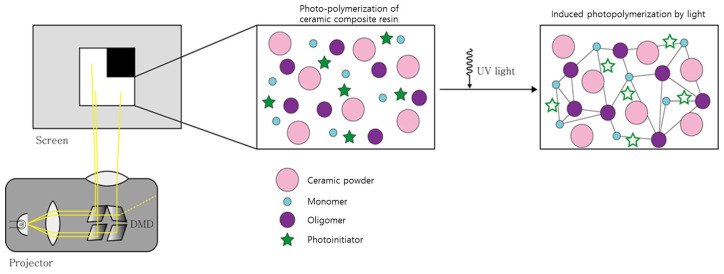
Photo-polymerization of ceramic composite resin.

The resin used in PP-type 3D printing is based on oligomers, monomers, and initiators, and absorbents and colorants are mixed together as needed [22,23]. As shown in Figure 1, when ultraviolet (UV) light is irradiated to the photocurable resin, the initiator reacts, and a free radical photo-polymerization reaction occurs. After that, oligomers and monomers are converted into polymers by chain formation, and the phase changes from a liquid state to a solid with shrinkage. At this time, the ceramic powder distributed inside the resin is surrounded and trapped by the solidified resin, forming the green body of the composite structure.

This photocuring process is based on the Beer-Lambert law. When UV light is transmitted vertically from the resin surface, light scattering and absorption processes occur. At this time, photocuring occurs to the depth where the amount of UV light penetrated reaches the curing threshold energy. As a result, the thickness of the cured polymer is called cure depth *C_d_*, and the formula for cure depth is shown in Equation (1) [24]:

(1) $$E_{c}=E_{o}^{-C_{d}/D_{p}},\;C_{d}=D_{p}\ln\left[\frac{E_{max}}{E_{c}}\right]$$

where *E_o_* is the light energy used for exposure, *E_c_* is the critical energy for curing, *D_p_* is the penetration depth of the specific light into the resin, and *E_max_* is the maximum exposure energy. In the case of a composite resin containing ceramic powder, the *D_p_* value is greatly reduced due to the light scattering of the powder surface and the decrease in permeability. Therefore, it is inevitable to increase the light irradiation energy to overcome the low photo-curability.

### 2.2. Fabricated 3D Printer

**Figure 2:**
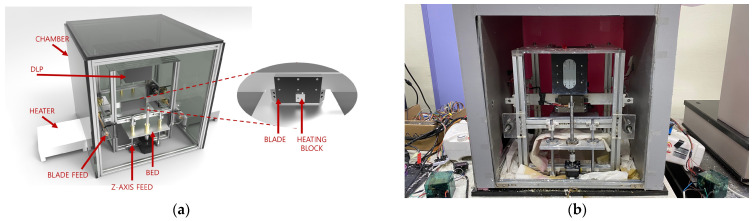
Developed DLP 3D printer: (**a**) CAD model; (**b**) fabricated DLP 3D printer.

High-viscosity composite ceramic resin has very low flowability, so it is difficult to supply materials smoothly and continuously with the bottom-up method, which is generally used in small PP 3D printers. Therefore, a newly designed 3D printer was developed for printing high-viscosity resins. The developed 3D printer was designed by forcibly blading by supplying material from the outside for each layer to the top-down printer structure. In addition, to minimize the influence of the external environment and control the process environment, as shown in Figure 2, all device structures necessary for 3D printing were placed in the insulation chamber and maintained at a constant temperature with a heater. The external chamber prevents degradation while performing a thermal insulation function using a heat-resistant EPS insulation board. For chamber heating, two 2 kW heaters (JY-901F, FUJI LIFE, Seoul, Republic of Korea) were attached to the outside of the chamber. The temperature was measured in real-time through a thermistor (NTC 10K 3950, Nitto Trading, Busan, Republic of Korea) installed inside, and the heater was controlled by a temperature relay (HAM3612, JENO, Busan, Republic of Korea) to maintain the set temperature.

**Figure 3:**
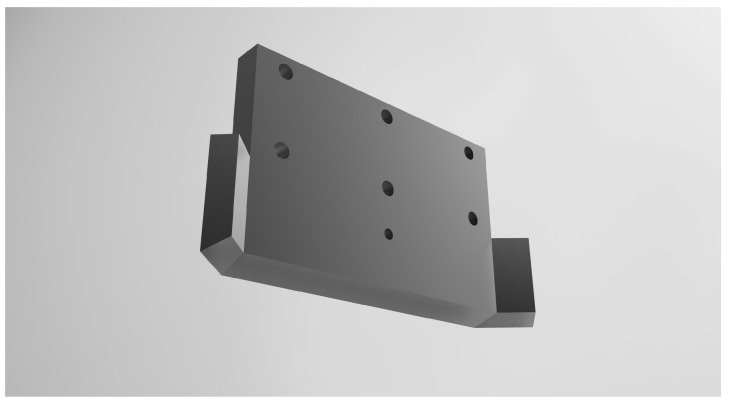
Blade shape.

The lab-made DLP 3D printer located inside the chamber consists of a DLP beam projector, bed, moving unit, and blade. The DLP beam projector (NVR, CARIMA, Seoul, Republic of Korea) was an FHD (1920 × 1080) class projector and has a pixel size of 50 µm when it has a build size of 96x54 mm. As the light source, a high-powered 405 nm wavelength LED was used to minimize heat generation. The output power was set to 80% of the maximum value for long-term stability. At this time, the light power measured by a UV light meter (UIT-250, USHIO, Tokyo, Japan) was 12.5 mW/cm^2^. The bed transfer part used a small DC step motor (42SHD0217-24B, Casun, Guangzhou, China) and driver (TMC2225, Makerbase, Guangzhou, China) with stable output and high resolution and was designed to move by 0.00125 mm per step by setting it to 32 micro steps. The blade was designed, as shown in Figure 3, to increase the fluidity and flatness of the supplied resin, and then two 30 W cartridge heaters were attached to control the temperature [25]. The overall equipment specifications are shown in Table 1.

### 2.3. Ceramic Composite Resin

**Figure 4:**
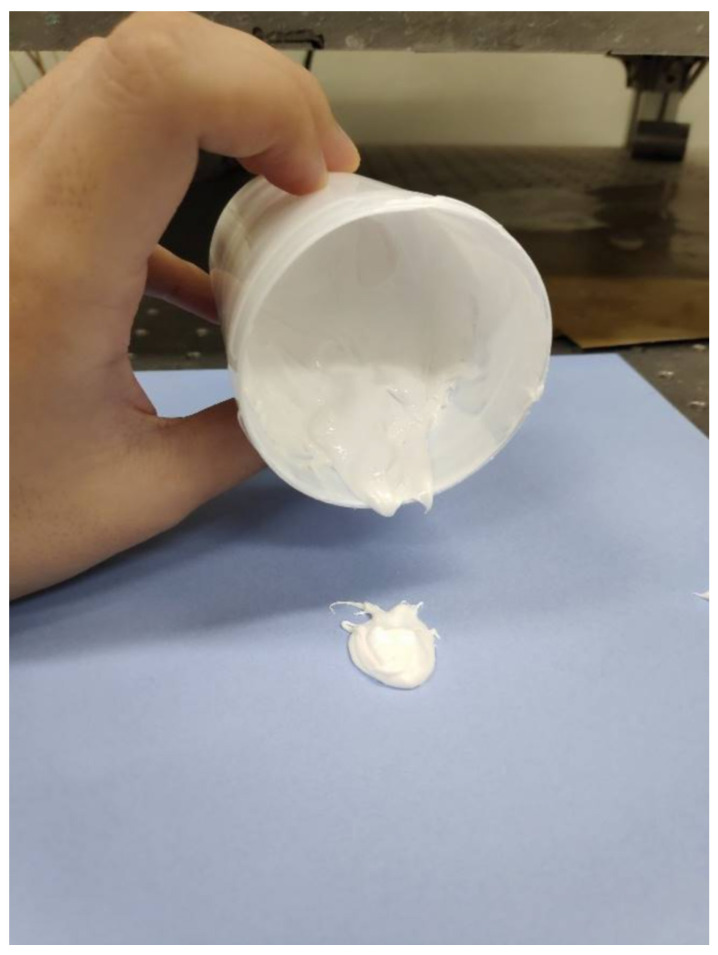
High viscosity/high contents zirconia composite resin.

The ceramic composite resin used in this experiment was the same composition developed in the previous study [21]. As shown in Table 2, the material used in the experiment was prepared by adding submicron tetragonal ZrO_2_ nanopowder (crystal size 40 nm) stabilized with 3 mol% Y_2_O_3_ (3YSZ, Tosoh Corporation, Minato-Ku, Japan) to the base resin mixed with monomer (HDDA, Sigma-Aldrich, St. Louis, MO, USA), photoinitiator (BAPO, Sigma-Aldrich, St. Louis, MO, USA), and dispersants (BYK-111, BYK, Geretsried, Germany). Vinyltriethoxysilane (VTES) coating was applied on the powder surface to increase the dispersion of the powder in the resin and delay the settling time. At this time, the viscosity of the resin was excessively high, so a small amount of butoxyethanol (Bu, Sigma-Aldrich, St. Louis, MO, USA) was added as a diluent to adjust the viscosity. The content of zirconia powder was fixed at 50 vol%, and the viscosity of the manufactured resin was 27,000 cps, which was almost non-flowable at room temperature, as shown in Figure 4.

### 2.4. Experimental Method

**Figure 5:**
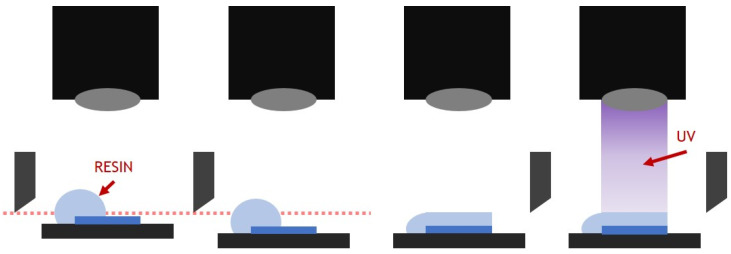
Printing process.

To evaluate and compare the properties of ceramic parts produced by the DLP 3D printing process, specimens were produced based on ISO test standards. Since zirconia is mainly used as a dental material, a 2.5t disk-shaped specimen of ∅15 mm was produced according to ISO 6872 Dentistry-Ceramic material. As shown in Figure 5, the printing process proceeded similarly to a typical top-down printer. The printed specimen was post-cured by a UV lamp for 2 h after cleaning the residue with isopropyl alcohol. To minimize the internal stress generated from the removal of solvents and residues, the post-cured green body was dried under a vacuum for 24 h at 25 °C. In order to minimize cracks caused by internal stress during sintering, after debinding at 300 °C for 3 h, heating was performed at 0.83 °C/min to 1450 °C. Finally, sintering was performed at 1450 °C for 3 h to perform grain growth and densification.

The fabricated specimens were initially observed visually, and then a contact-type surface profiler (SJ-400, Mitutoyo, Kawasaki, Japan) was used to quantitatively measure the degree of warpage. In order to observe internal microcracks, the specimens were notched, and then the cross-section of the specimens was observed by scanning electron microscopy (SEM) (VEGA3 LM, TESCAN, Brno-Kohoutovice, Czech Republic) through impact fracture. In order to measure the densification of the printed specimen according to process changes, the relative density was measured using the Archimedes method. For this, a density measuring instrument using a precision scale (CBL-320, CAS, Yangju, Republic of Korea) was made, and the measured value was substituted into Equation (2) to calculate the density (*ρ*).

(2) $$\rho =\frac{W_{a}}{W_{a}-W_{l}}\times \left(\rho_{l}-\rho_{a}\right)+\rho_{a}$$

where *W_a_* is the weight of the sample in the air [g], *W_l_* is the weight of the sample in liquid [g], *ρ_l_* is the density of liquid [g/cm^3^], and *ρ_a_* is the density of air [g/cm^3^]. The theoretical density of the green body before sintering was calculated to be 3.545 g/cm^3^ based on the material ratio. The theoretical density of the sintered body was set to be 6.08 g/cm^3^, which is the density of pure zirconia.

A micro Vickers hardness tester (FM-800, Future-Tech, Kawasaki, Japan) was used to evaluate the mechanical properties of the finally sintered specimen. The indentation load was set to the maximum value of the tester, 2 kgf, and the indentation time was set to 80 s, and the hardness was calculated by observing the indentation marks.

## 3. Results and Discussion

### 3.1. Blade Temperature

When using a high viscosity resin of 27,000 cps as used in the experiment, there was little fluidity at room temperature, and agglomeration between materials was severe, making it impossible to planarize with a general blade. Since the viscosity of a material is generally inversely proportional to the temperature, a heating blade was designed to instantly increase material flowability. In this case, when the high-viscosity composite resin temporarily contacted the heated blade, the viscosity of the resin was lowered so that planarizing was possible. However, since excessive temperature could cause changes in material properties, the flatness of the resin was visually examined according to the blade temperature. The blade temperature was varied from 15 °C, which corresponds to room temperature, to heated 40 °C, 70 °C, and 100 °C.

**Figure 6:**
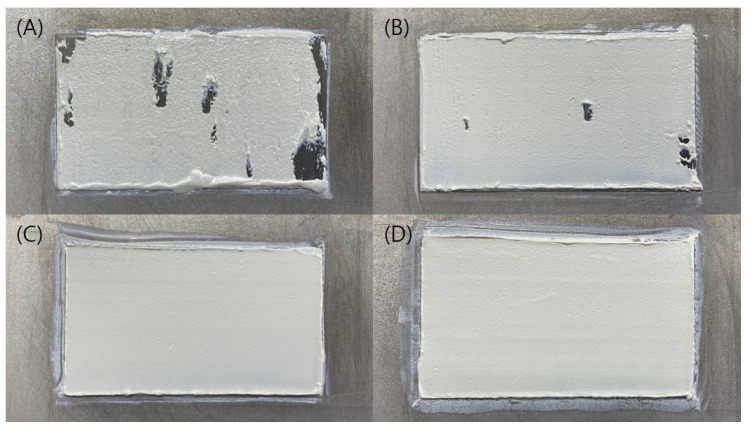
Flatness according to various blade temps: (**A**) 15 °C; (**B**) 40 °C; (**C**) 70 °C; (**D**) 100 °C.

As shown in Figure 6, in the case of an unheated 15 °C blade at room temperature, when planarization was performed, materials were agglomerated due to the viscosity of the resin, making it impossible to planarize smoothly even after the repeated process. When the blade temperature was increased by 40 °C, resin planarization was possible to some extent as the material agglomeration was reduced, but an application defect was still observed as resin with low flowability was agglomerated in some areas. On the other hand, when the blade temperature was raised to 70 °C or higher, it was confirmed that the planarization was possible without a defective area, unlike the previous results. However, as the temperature increased, the thermal effect on the resin also increased, and rapid vaporization of the added solvent might occur, so in this experiment, 70 °C, which was capable of proper layer planarization, was selected as the blade temperature.

### 3.2. Chamber Temperature

To minimize the thermal impact caused by the high-power UV light applied for curing and to secure the reproducibility of the experiment, a chamber was made, and the effect was observed while varying the internal temperature. The chamber temperature was varied by heating from room temperature of 15 °C to 40 °C and 60 °C. Process conditions other than the chamber temperature were fixed at a blade temperature of 70 °C and an exposure time of 1 s.

**Figure 7:**
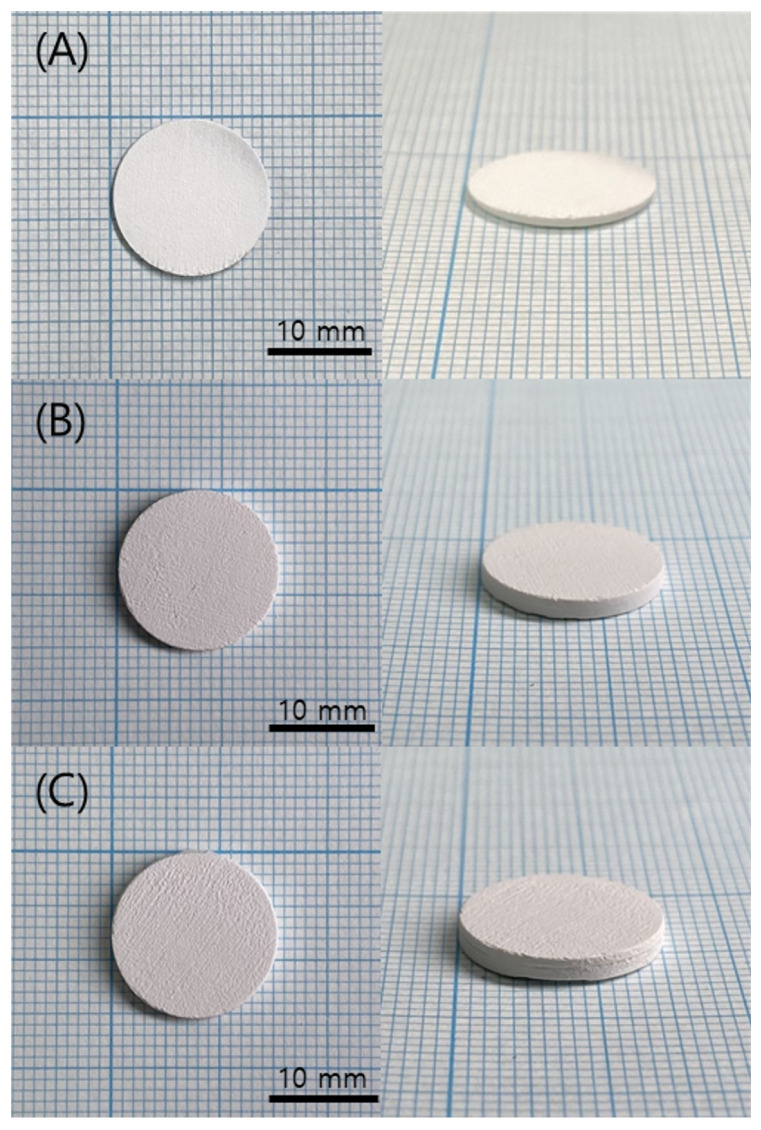
Green body specimens according to various chamber temps: (**A**) 15 °C; (**B**) 40 °C; (**C**) 60 °C.

**Figure 8:**
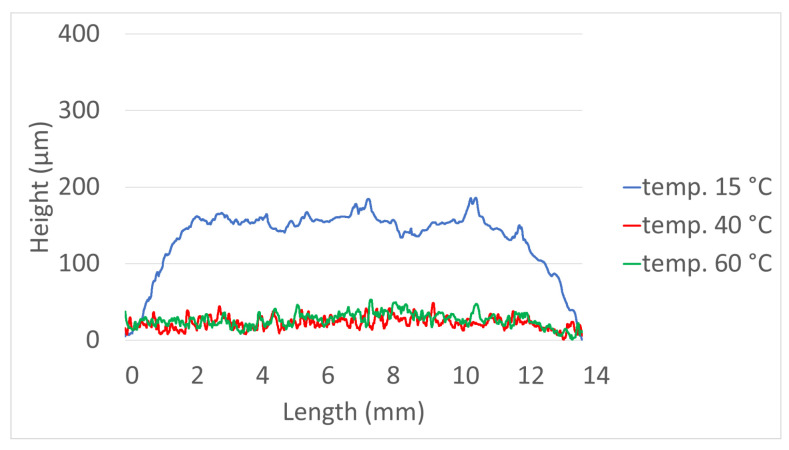
Surface profile—Green body specimens according to various chamber temps.

**Figure 9:**
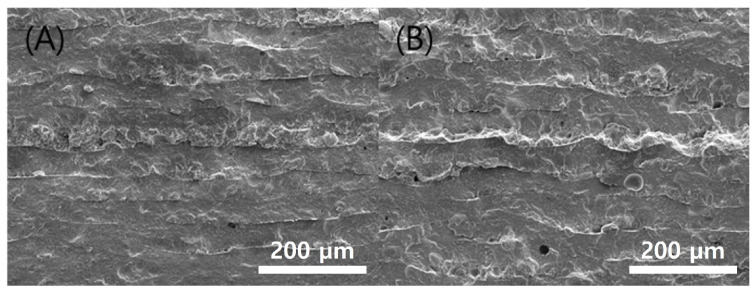
Cross-sectional SEM images green body specimens according to various chamber temps: (**A**) 40 °C; (**B**) 60 °C.

The results of the printed specimens at each chamber temperature were shown in Figure 7. At an internal temperature of 15 °C without heating the chamber, warpage often occurred after curing due to the difference between the heat generated during DLP exposure and the ambient temperature. Accordingly, as the printing was repeated, the internal stress due to the warpage accumulated, causing the edges to lift. Due to this phenomenon, the blade was caught on the edge of the specimen attached to the bed, causing specimen damage and making additional printing impossible. This was also clearly observed in the surface profile data shown in Figure 8. It was confirmed that the edge of the specimen was lifted about 200 µm due to the warpage phenomenon. On the other hand, the warpage could not be observed visually when the chamber temperature was 40 and 60 °C, and it was confirmed that the flat disc specimen was manufactured without any abnormality in the surface profile data. In addition, as shown in Figure 9, defects such as delamination or cracks between printed layers could not be observed even when the printed cross-section was observed by SEM.

**Figure 10:**
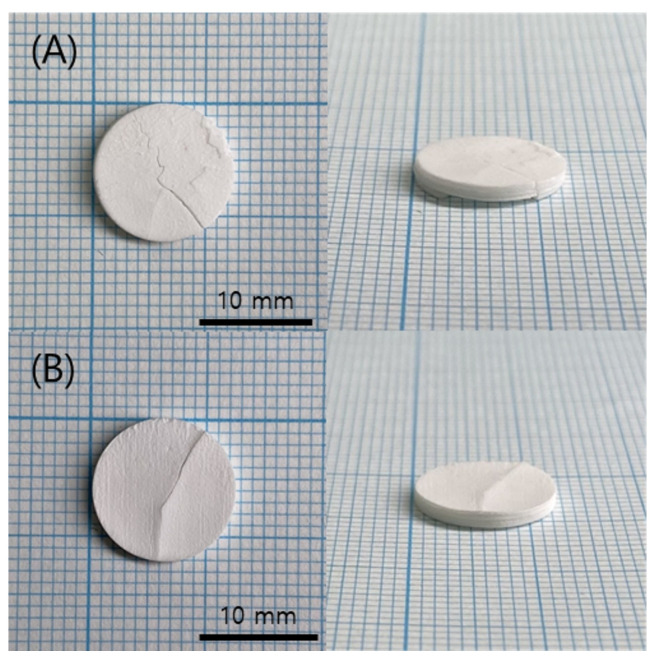
Sintered specimens according to various chamber temps: (**A**) 40 °C; (**B**) 60 °C.

**Figure 11:**
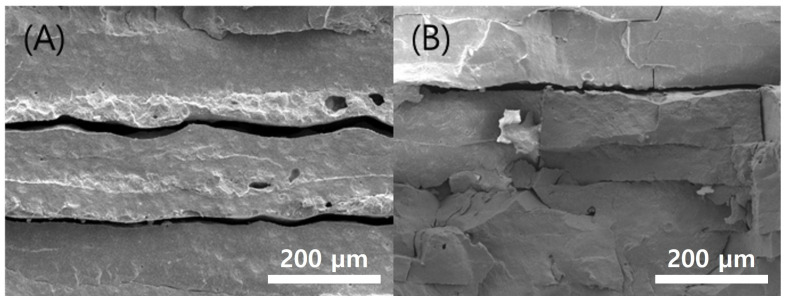
Cross-sectional SEM images Sintered specimens according to various chamber temps: (**A**) 40 °C; (**B**) 60 °C.

Sintering was performed on the green body specimen, which was obtained by the normal completion of printing at the chamber temperatures of 40 °C and 60 °C. No problems were observed in the green body state before sintering, but cracks occurred on the surface of both specimens during the sintering process, as shown in Figure 10. In addition, cross-sections of each specimen were observed by SEM, and it was found that delamination occurred between the printed layers, as shown in Figure 11. This means that the structural stability and uniformity of the specimen were not sufficiently secured in the green body state, resulting in slight warping and cracking during the high-temperature debinding and sintering process.

In a general photocuring process, the strength and structural stability of the specimen is proportional to the light power and exposure time. However, in the commercial photo-polymerization process, it is common to perform long-term post-curing after curing with minimal light energy to improve printing speed and precision. However, in the case of the composite resin containing ceramic powder used in this study, the light transmittance was remarkably low, so the resin inside the structure could not be post-cured when printing was completed. Therefore, if light power was not high enough during the printing process to cure completely, defects might occur due to fine uncured areas during debinding and sintering. Therefore, a sufficient amount of light energy irradiation was required during the printing process [26,27].

### 3.3. Curing Time

As shown in the previous experimental results, in order to minimize defects such as specimen cracks during sintering, green body specimens should be fabricated through a sufficient curing reaction so that no uncured areas exist. Since the light power was increased as much as possible considering the lifetime of the used DLP, the exposure time was changed to induce a sufficient curing reaction. The exposure time was set to 0.8 s, 1 s, and 1.6 s, respectively, and the cured depth was measured to be 80 µm, 100 µm, and 120 µm in each case. For other process conditions, a blade temperature of 70 °C and a chamber temperature of 40 °C were used, which could increase stability and minimize thermal damage to the resin during printing based on the previous experimental results.

**Figure 12:**
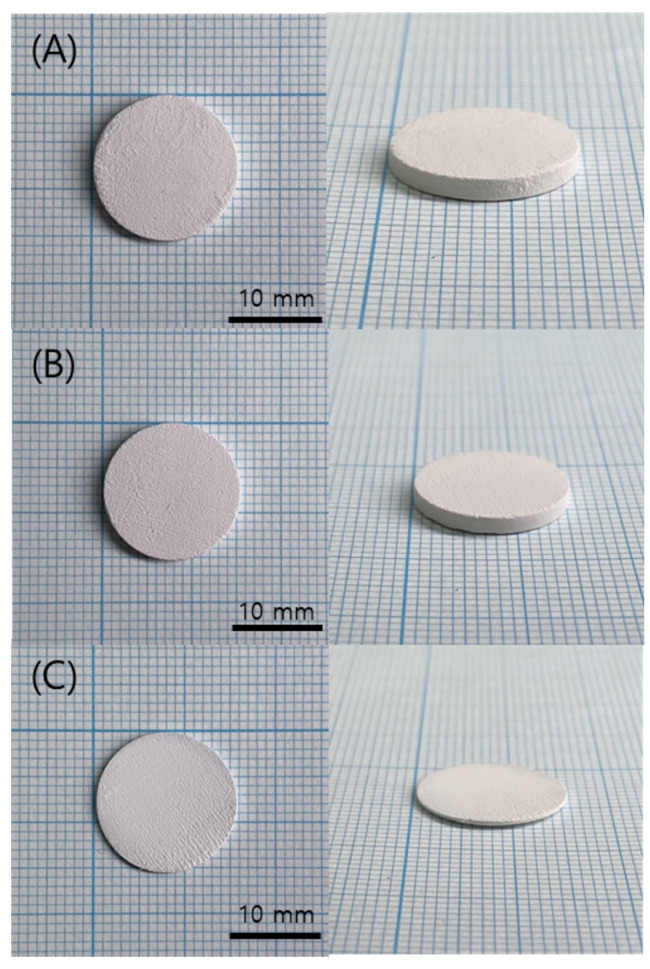
Green body specimens according to various curing times: (**A**) 0.8 s; (**B**) 1 s; (**C**) 1.6 s.

**Figure 13:**
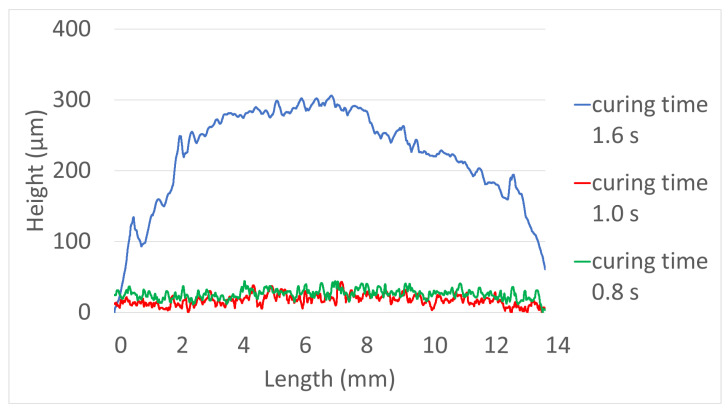
Surface profile—Green body specimens according to various curing times.

**Figure 14:**
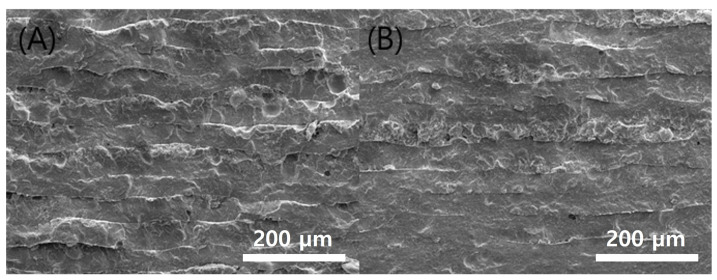
Cross-sectional SEM images green body specimens according to various curing times: (**A**) 0.8 s; (**B**) 1 s.

When the curing time was set to 1 s or less, no visible cracking or warping was observed in the printed green body specimens, as shown in Figure 12. However, when the exposure time was set to 1.6 s for sufficient curing, edge distortion due to warpage was observed, as shown in Figure 13, which stopped the printing process. As shown in Figure 14, when the curing time was 1 s, no significant abnormalities were observed in the cross-section of the printed specimen. However, when the curing time was reduced to 0.8 s, the boundary between layers was observed more clearly due to the uncured area.

**Figure 15:**
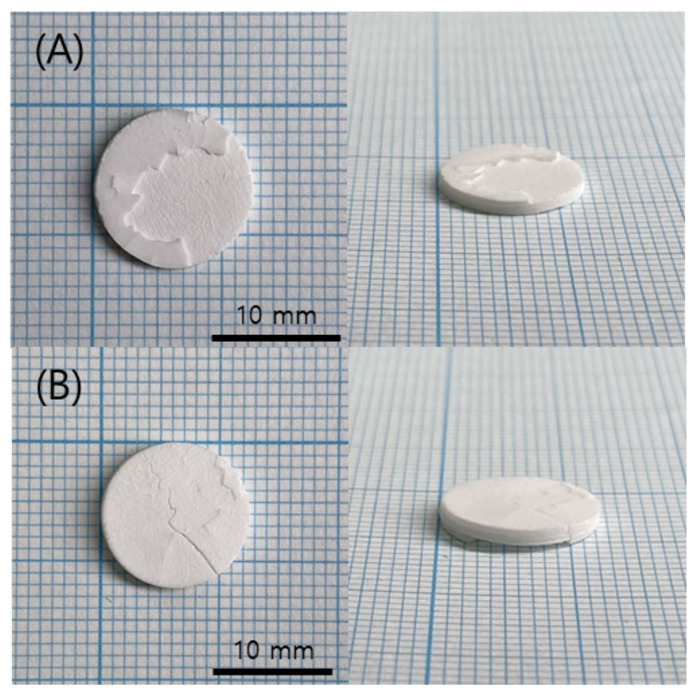
Sintered specimens according to various curing times: (**A**) 0.8 s; (**B**) 1 s.

Sintering of specimens cured with 0.8 s and 1 s, respectively, the sintered results were similar to those of the previous experiment. Although no cross-sectional and appearance defects were observed before sintering, both conditions lacked sufficient curing and structural stability in the green body state due to insufficient exposure energy during printing, resulting in multiple cracks during sintering, as shown in Figure 15. In particular, for the 0.8 s specimen with a relatively short exposure time, not only surface cracks were observed, but also areas where the entire layer delaminated during sintering.

### 3.4. Curing Pattern

**Figure 16:**
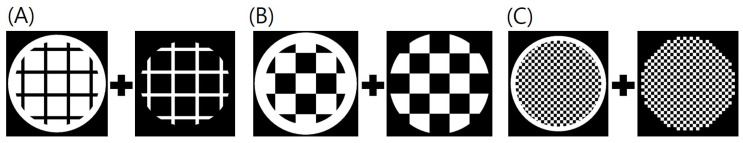
Patterned curing images: (**A**) Square and line pattern (3 × 3 mm square and 0.1 mm line pattern); (**B**) big square pattern (3 × 3 mm square pattern); (**C**) micro square pattern (0.5 × 0.5 mm square pattern).

Therefore, in order to suppress the generation of cracks during sintering, it was necessary to produce green body specimens with minimized uncured areas by irradiating more exposure energy. For this, a long exposure time of 1.6 s or more must be irradiated, but as shown in the previous experiment, in this case, warpage was generated due to excessive heat of UV light and curing shrinkage, making the printing process impossible. Therefore, during the printing process, the entire area of each layer was not cured at once. Instead, it was divided into patterns, as shown in Figure 16, and exposed in two steps. In this case, it was expected to reduce the phenomenon of strong shrinkage caused by high energy during photocuring, similar to how the powder bed fusion (PBF) printing process achieves effective cooling by irradiating the laser in divided zones to disperse the heat generated during the process [28,29].

The proposed pattern shapes were all designed to disperse the shrinkage stresses that occur when the entire area was cured at once. In the case of the pattern in Figure 16A, the light was irradiated to most of the area during initial curing, but since each modeling area was not connected to the other, it was designed in a way to suppress simultaneous shrinkage of the entire area. Then, the area corresponding to the uncured area was additionally exposed in a 0.1 mm linear pattern to cure the entire area. Next, the entire area was divided into halves to create a chessboard-like shape, as shown in Figure 16B. Even in this case, each cured area was not connected as much as possible to minimize shrinkage stress by simultaneous curing. Lastly, the micropattern shape was designed as shown in Figure 16C, focusing on the fact that the warpage decreases as the curing area decreases. The area of each micropattern had an area corresponding to about 2.8% of the aforementioned big square pattern. Process conditions other than the pattern shape were set to 1.6 s of exposure time, chamber temperature of 40 °C, and blade temperature of 70 °C for sufficient curing.

**Figure 17:**
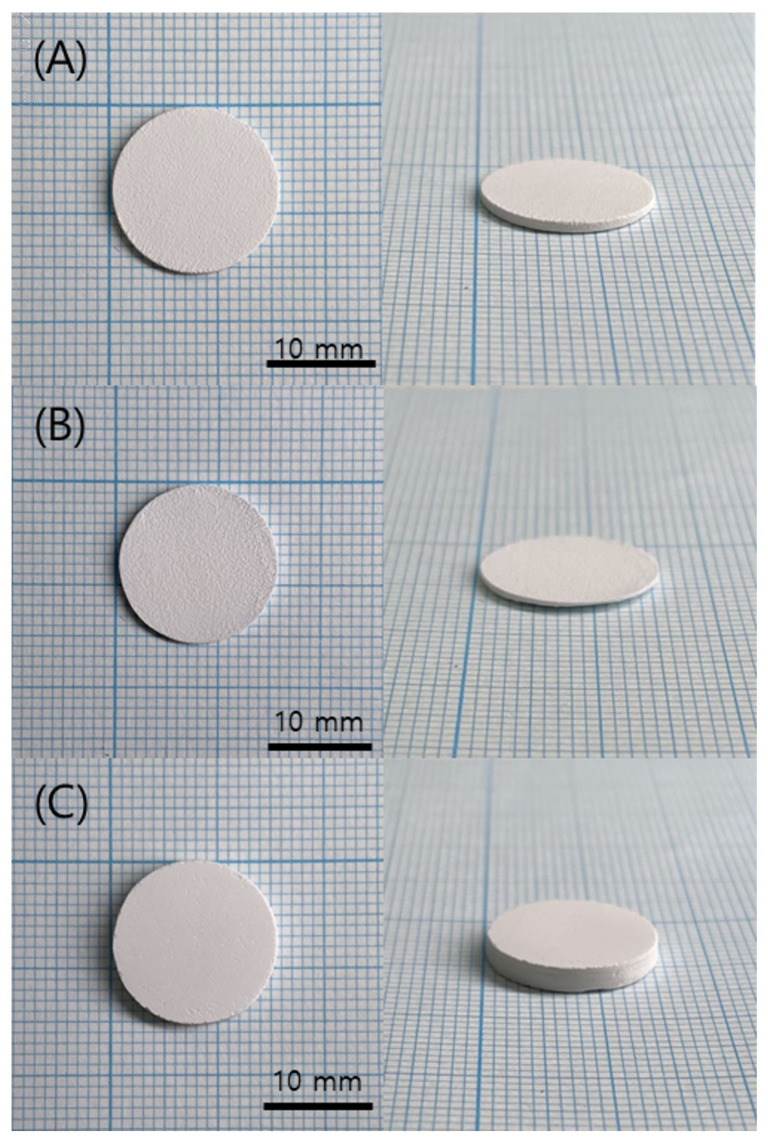
Green body specimens according to various pattern types: (**A**) square and line pattern; (**B**) big square pattern; (**C**) micro square pattern.

**Figure 18:**
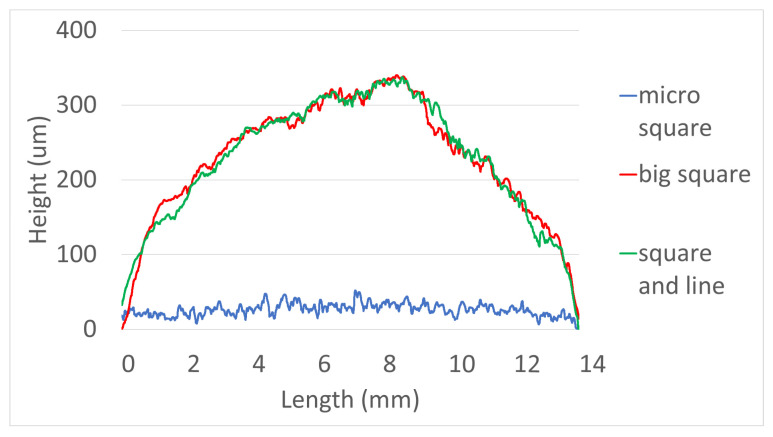
Surface profile—Green body specimens according to various patterns.

**Figure 19:**
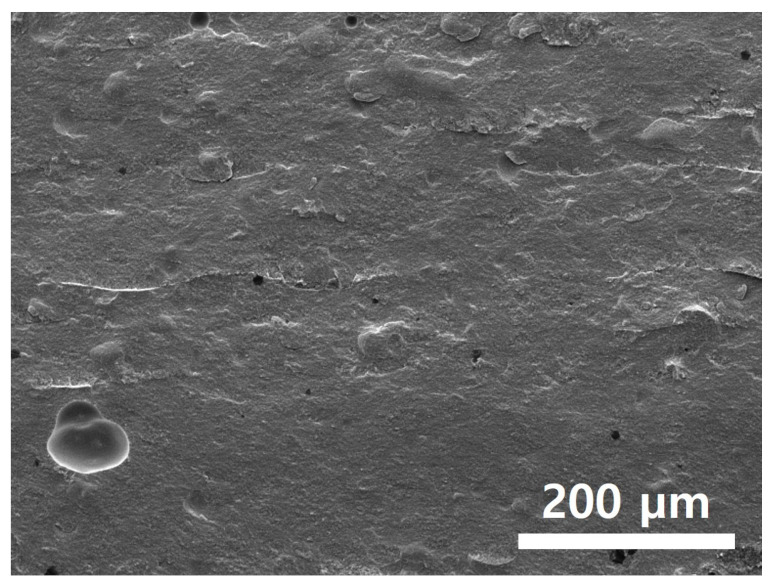
Cross-sectional SEM image of micro square pattern.

The printed specimen for each pattern was shown in Figure 17. For the square and line pattern case, the warpage seemed to improve at the beginning of the printing, but as shown in Figure 18, the warpage gradually increased as the printing progressed, eventually stopping the process. This phenomenon also occurred in the big square pattern. In other words, the above two patterns provided some improvement compared to curing the entire area at once, but it did not become a fundamental solution. However, in the case of the micro square pattern, in which the area of each pattern was drastically reduced, it was possible to print without any singularities or warpage in appearance, even though sufficient curing time was irradiated. In addition, as shown in the cross-sectional SEM in Figure 19, the boundary between layers or between patterns was hardly observed, so it was confirmed that the uncured area was minimized through sufficient light energy irradiation.

**Figure 20:**
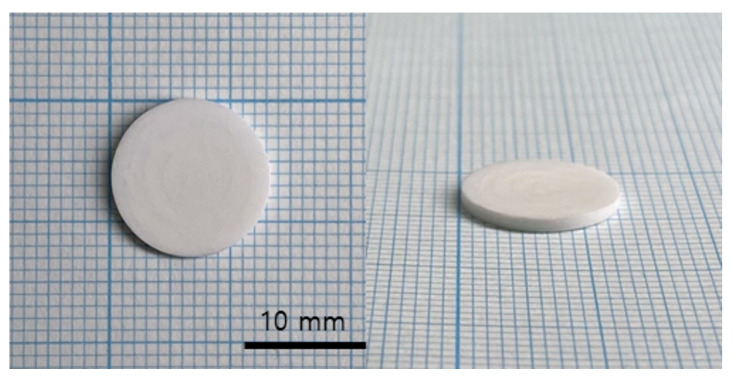
Sintered specimen of micro square pattern.

**Figure 21:**
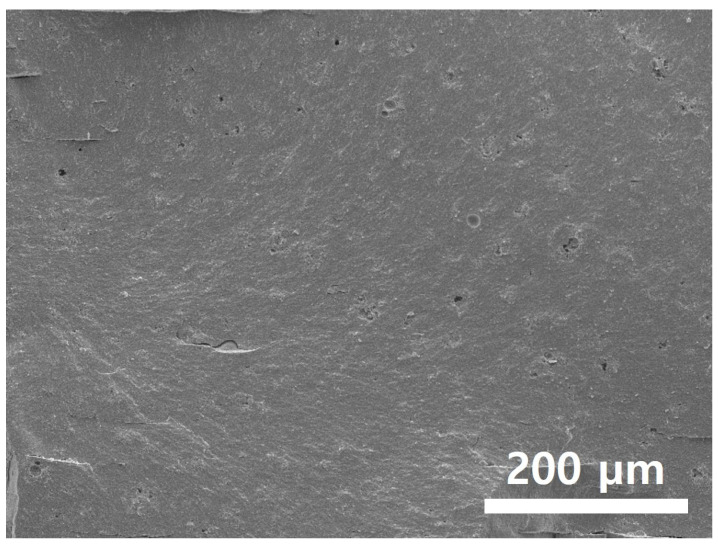
Cross-sectional SEM image of micro square pattern after sintering.

As a result of sintering the micro square pattern specimen, which secured structural stability through sufficient curing energy irradiation, as shown in Figure 20, a disc specimen without cracks could be obtained. Additionally, as shown in Figure 21, it was confirmed that the specimen was properly sintered without any layer delamination or cracks through the cross-section SEM. Accordingly, in order to induce a sufficient curing reaction of the high-viscosity zirconia composite resin while minimizing warpage, it was determined that it was appropriate to cure the zirconia composite resin for 1.6 s with a micro square pattern of 0.5 × 0.5 mm while maintaining the blade temperature of 70 °C and the chamber temperature of 40 °C.

## 4. Analysis of Specimen

### 4.1. Relative Density

In order to check the density of the printed specimens, the relative density was measured based on the theoretical density using the Archimedes method. In cases where the printing was stopped due to warping, the density measurement was not possible due to the severe warping and in-process damage of the specimen, so the measurement was performed only on the completed specimen without warpage. Three different specimens for each condition were repeatedly measured three times. 

The relative density of the specimen in the green body state before sintering was not significantly different for each process condition, as shown in Table 3. In particular, there was little difference according to the internal chamber temperature. However, there was a slight difference for each condition depending on the curing time, and in particular, when curing with 1.6 s using a micro square pattern, it was observed that the relative density increased. This means that if the chamber temperature was maintained above a certain level, the internal densification and curing of the specimen were not significantly affected, but the exposure time had a relatively large effect on the densification of the specimen. On the other hand, in the case of the sintered specimen, the difference occurred depending on whether or not cracks occurred. That was, it was observed that the specimens which were cured for less than 1 s developed cracks during the sintering process, leading to a significant reduction in relative density. On the other hand, the specimen cured with 1.6 s using the micro square pattern had a relatively high relative density of 95.77%.

### 4.2. Vickers Hardness

To compare the mechanical properties of specimens printed with each process condition, Vickers hardness was measured. In the case of the printed green body specimen before sintering, it was impossible to measure the Vickers hardness because the hardness was too low since it basically had the characteristics of plastic. Only the sintered specimens were measured for Vickers hardness with an indentation load of 2 kgf and an indentation time of 80 s. In addition, three specimens were tested for each process condition to obtain the average and standard deviation, as shown in Table 4.

The surface hardness values were different depending on the exposure time. This was because the uncured area increased when the exposure time was short, and thus the microcracks during sintering also increased. These microcracks eventually cause a decrease in surface hardness. Therefore, when curing was performed for 1.6 s with the micro square pattern capable of minimizing the uncured region and ensuring structural stability, a surface hardness of 12.2 GPa, which is almost equivalent to the Vickers hardness of commercial zirconia, could be secured. On the other hand, it was confirmed that the internal chamber temperature had no significant effect on the mechanical properties of the final product if it was maintained above a certain level.

## 5. Conclusions

In this study, a 3D printer equipped with a heating blade and a heating chamber was fabricated, and a new pattern-shaped curing was proposed to enable high-quality PP-type 3D printing using high-viscosity zirconia composite resin. First of all, for the smooth application of the high-viscosity composite resin, it was necessary to reduce the viscosity of the resin temporarily during the planarization process, and a temperature-adjustable blade was used for this purpose. It was found that a blade temperature of about 70 °C was required to flatten a high-viscosity ceramic composite resin of about 25,000 cps with minimal resin damage. In addition, it was confirmed that if the temperature of the internal chamber during the process reached 40 °C or higher, the warpage of the specimen due to heat during printing could be improved. On the other hand, it was confirmed that the additional improvement effect was insignificant even if the temperature was further increased above 40 °C.

The process factor having the greatest influence on the quality and mechanical properties of the printed part before and after sintering was the total amount of irradiated energy directly related to the uncured area. In this study, the maximum power was limited considering the lifetime of the DLP projector used, so the total irradiation energy was changed by adjusting the exposure time. The quality and mechanical properties of the printed specimen before and after sintering increased in proportion to the exposure time, but as the exposure time increased, excessive shrinkage due to heat and phase transition accompanied the warpage, making it impossible to perform the proper printing process. To solve this problem, a newly proposed micro square pattern-based curing method was applied, and a structure without warpage could be produced even with an exposure time of 1.6 s. Through this, in this paper, it was possible to fabricate a pure zirconia printed part with a relative density of 95.77% and a Vickers hardness of 12.20 GPa after sintering. Considering that the theoretical hardness value of zirconia is 12 to 12.5 GPa, and the hardness of previous 3D printed zirconia specimens is also 12 to 13 GPa, it was confirmed that the 3D printed parts fabricated based on the proposed process from this study secured the physical properties that could replace commercial zirconia parts. Ceramics, including zirconia, have unique characteristics such as high strength and heat resistance, so they are expected to be actively applied to bio-industries such as artificial teeth or bones as well as mechanical parts. Therefore, through this study, it is expected that the development of the 3D printing process of high-viscosity ceramic composite resin for the PP method, which can produce high-quality and complex shapes, can be used in various ways for the fabrication of ceramic parts with precise shapes.

## Figures and Tables

**Table 1:** Specification of fabricated DLP 3D printer.

Specification	Value
Build size	96 × 54 × 30 mm
DLP pixel resolution	50 µm
DLP UV power	12.5 mW/cm^2^ at 405 nm
Layer thickness	50 µm

**Table 2:** Compositions of ceramic composite resin.

Material	Volume Ratio
Ceramic (ZrO_2_)	50%
Monomer (HDDA)	42.5%
Photoinitiator & Dispersant & Solvent etc.	7.5%

**Table 3:** Relative density.

	Curing Time: 0.8 s Chamber Temp: 40 °C No Pattern	Curing Time: 1 s Chamber Temp: 40 °C No Pattern	Curing Time: 1 s Chamber Temp: 60 °C No Pattern	Curing Time: 1.6 s Chamber Temp: 40 °C Micro Square Pattern
Average relative density before sintering (standard deviation)	97.22% (0.07789)	97.64% (0.04321)	97.60% (0.02494)	98.51% (0.04546)
Average relative density after sintering (standard deviation)	92.05% (0.1551)	92.66% (0.3894)	92.57% (0.08577)	95.77% (0.09286)

**Table 4:** Vickers hardness.

	Curing Time: 0.8 s Chamber Temp: 40 °C No Pattern	Curing time: 1 s Chamber Temp: 40 °C No Pattern	Curing Time: 1 s Chamber Temp: 60 °C No Pattern	Curing Time: 1.6 s Chamber Temp: 40 °C Micro Square Pattern
Average HV_2_ after sintering (GPa) (standard deviation)	8.75 (0.04714)	10.99 (0.09092)	11.17 (0.31188)	12.19 (0.15063)

## Data Availability

Not applicable.
